# Popular Medical Education; Its Aid in Limiting the Spread of Disease1Read at the annual meeting of the Gross Medical Club, January 23, 1903.

**Published:** 1903-05

**Authors:** J. D. Macpherson

**Affiliations:** Akron, N. Y.


### BUFFALO MEDICAL JOURNAL.

VOL. XLII.—LVIII. MAY, 1903. No. 10.

### ORIGINAL COMMUNICATIONS.

### Popular Medical Education; Its Aid in Limiting the Spread of Disease.[1]

By J. D. MACPHERSON, M. D., Akron, N. Y.

The night of fable and the twilight of conjecture.
What was yesterday a theory, is today a condition.

PRIMITIVE man in his rude tepee, as he ministered to his loved one in its bark hammock and watched with unavailing eye the ravage of disease in the flicker of his uncertain firelight, paused often in his comfortless vigil to invoke the aid of the Great Spirit for the staying of the malady which came and which worked in form insidious and unknown to him. At once father and physician, as he threw back at dawn the flap of his tent, the grey light of morning showed him that death's relentless hand had taken as his toll the child and patient. What must have been the overwhelming sense of helplessness that came to him? He was fit for meeting the contingencies of life upon every level except that of disease. His strong arm might protect the inmates of his home against the foemen; his intelligence secured for him food in stream and forest; instinct blazed before him the path of duty and cleaved for him the separation between right and wrong.

But here was he at bay. The smiting plague was he impotent against. He beheld effects; of causes he was ignorant. Disease was to him an ambushed foe; the source or direction from whence flew the poisoned barbs was to him unknown. And yet he had good ideas of symptoms and was in his way an expert diagnostician. Exanthemata upon other members of his family or his tribe were as patent to him as the heaven lowering rain or

1. Read at the annual meeting of the Gross Medical Club, January 23, 1903.

the track of wild animal in the chase. The hectic flush, the wasting form, the fever lit eye, the extravagances of delirium—all these were to him as a tale that is told. But the tale had always the one and only ending. Ever the silver cord was loosed, and the golden bowl was broken. Man set out to his long home, and the fierce tide of the battle charge or the calm deliberations of the council circles knew him no more. The Evil Spirit had conquered.

Small wonder, then, that we see this man of the world's youth time, turning from his circumscribed realm of the seen and seeking far afield the ministry of the supernatural. Small wonder that he strove with all that in him was to go beyond the short tether of sense, in the invoking of spirit's aid and the offering of sacrifice in the form of treasure. It was the age of charm and amulet, the weird music of the incantation of the sorcerer; the era of the medicine man, remedial with roots and herbs.

Ancient history is full of the ravages of plague. In the Iliad of Homer we behold the army of the Greeks devastated by disease and halting for weary months to propitiate the angry gods. Hecatombs of kine and of sheep ascend from the kindled altars in smoke and savor of burning flesh, while the air is laden with the breath of incense.

Yet even in those fragrant days of a storied past the restless mind of man was seeking, as for hidden treasure, the knowledge of the dread bar to human progress in the guise of disease. Man gropes, but he gropes persistently. If he hopes ever so faintly, yet doth he still hope. And it has been this hope in the heart that through all time has nerved him to the seeking out of the hidden cause of things. Disease was the manifestation, symptoms the phenomena, of something behind the opaque wall of the seen. In the past, the ministry of medicine has been largely to the shadowy manifestation and humanity has made the prayer of Ajax its own, that it might have light if only to see its foeman's face.

This has come with the perfecting of the oil immersion lens. This lens gathering up the waves of light has focussed them upon the lurking places of hidden cause. It has been reserved for Nineteenth Century man to get the first straight look at the arch enemy of mankind. The data he has thus collated has been correlated into a science by itself. Bacteriology is the one thing that makes prevention of disease possible. A glass of water is found to contain creatures more deadly in their potentiality than the huge, unwieldy beasts of the Mesozoic.

The microscope shows that the great foe to human life lives; that it is an entity. Like ourselves, it has a birth and a death. To survive, it must be fed. To grow and thrive its habitat must be rich for its nourishment. To sterilise the habitat, therefore, makes the death of this elusive being surer and reduces propagation of the species to a minimum. With these facts learned, the science of prevention opens before us a realm immeasurable in extent and limitless in suggestive possibility. Our profession has not been slow in entering upon this rich heritage. But the great battle has only begun. With the enlarged vision of our microscopic eyes, we have discovered, it is true, the omnipresence of our foes. And now, like true generals, we must post our sentinels upon each coign of vantage. Our search lights must seek out the hidden things of field and trench, of woodland and hillside, of sea and air. For the mighty hosts of bacteria swarm by the countless millions, using every means of transportation from the tiny atom for an air ship, to the giant ocean liner. Our foes are small and, in order to sweep clean, our brooms must be of fine quality and of incessant and assiduous use. Expert clinicians in the field, exact experimenters in the laboratory, a definite knowledge of the habits of bacteria will soon tell upon the mortality claimed by disease. When Edward VII. was crowned to his kingly station, there was crowned likewise a man behind the throne in the person of Lister, the father of antisepsis.

The tendency of all knowledge is toward synthesis. The medical science is no exception to this rule. Diseases to which have been given their appropriate and almost countless nomenclature, will be found on last analysis oftentimes to be caused by the same germ working in different habitat.

That knight-errant of modern medicine, Professor Koch, of Berlin, has by analysis and grouping, following accurate experimentation in the laboratory, resolved the old time lupus into but one of the many manifestations of tuberculosis. Scrofula, likewise, has been thus reduced to its common denominator. The medical profession has now a firm grasp, that is ever growing firmer, of this great fact. What is now needed is the education of the masses in order that their intelligent coöperation shall be obtained.

That they will give this coöperation when once the field has been opened to them, is seen in the manner in which the farmer attacks the parasites invading his orchard and his grain field. He has learned from the experiments of our surpassing agricultural stations how best to attack these foes of the husbandman. These are the clinical laboratories from which come the expert knowledge that builds an exact science, and which spreads through the medium of farm journals, containing rules for diagnosis and the appropriate treatment for each individual pest. The wide dissemination of such literature among the intelligent and reading agricultural classes, has made the entire country conversant with the process of extermination. If farmers can be taught to preserve their crops, they can also be taught to follow the laws of health. These laws may be codified in three great departments,—hygiene, sanitation, and therapeutics. The first two of these are for all mankind; the third is the realm of the scientific medical practitioner.

We who were born in the past century should bequeath to our children a better physique, a more enduring constitution and better mental capacities, than possessed by the generation just passed. Life becomes more and more strenuous, competition more and more keen, and the law of the survival of the fittest will receive clearer emphasis in the future than in the past. It follows, therefore, that a life of larger demands necessitates a better bodily machine and consequent superior mental equipment. A proper knowledge of the needs of the physical system and the safeguards for its healthful maintenance should, at the age of 15, in this generation, be a more real possession than it was at 50 in the past generation. Our immediate descendants should instinctively select that for which we had to delve. They should carve what we have hewn. The first essential in prevention of disease is that which shall upbuild the whole man with proper physical endowment, on the principle of a sound mind in a sound body.

Sanitation should be so perfect as to make it impossible for an unsanitary home to exist from the standpoint of public opinion alone. But it has ever been true that what is everybody's business is nobody's business, and public opinion is powerless unless crystalised. It should, therefore, be the province of the state that upon the appearance of an unsanitary home in a given neighborhood, there should be the same procedure as at the outbreak of diphtheria or scarlet fever. Such home should be quarantined at once and the sanitary office, through its appropriate representative should afford an immediate and effective cleaning. Tenement districts should be inspected by district physicians, and sanitary inspectors should be provided whose vigilance should be severe and unceasing. The recent cleansing of the Cuban cities by General Wood, shows what can be done by a corps of sanitary engineers working under the surveillance of strict military régime. Some of the effectiveness of the military idea might properly be carried over and applied by the health officers under civil law. In Havana, formerly a center where yellow fever was indigenous, yellow fever no longer exists, because its native habitat has been sterilised through the employment of proper sanitary measures. Compulsory laws will, in one decade, start a community on the road to a condition where sanitation is the rule, not the exception.

The recent enactment of laws against expectoration in many of the cities of the country, is one of the powerful agencies directed against the propagation of tuberculosis and reveals, as in a flash, to the spitter the reason why he should not spit. This is but suggestive of the time when our alleys and sewers shall be flushed with antiseptics. The invasion of deadly gases in cities, whose plumbing is constantly improving, has been reduced to a minimum. The use of the incandescent light in places hitherto dark and the native breeding places of germ propagation, has opened their crannies and recesses to the beneficent action of sanitary endeavor. The large improvement in ventilating systems will also cause great changes to occur in the architecture of the future. Fresh air and sunlight, and electric light as well, have proven themselves to be the most direct enemy furnished as yet for the destruction of bacteria. Linked to these agencies, scientific hygiene and sanitation form the real key to the active propaganda of limiting disease.

The application of this hygiene should begin in the home. Here, as in all things else, the home is the real fountain. Whether this source shall be contaminated or not, depends upon the popular appreciation of the science of prevention. The physical environs of the home should have first consideration.

If the sanitary conditions in large cities were half as bad as exist in the rural sections of the country, the death-rate would become equal to that in time of plague. City people oftentimes come out to the country to obtain fresher air and purer physical environs, than to the old time idea exist in cities.

But do they always get these things? Many times they come to an old homestead very picturesque amid its foliage of overshadowing evergreens. Here, by all the conventions of the past, a convalescent should be charmed back to health and contentment. But of late the gloom of sickness has detracted from the beauty of the landscape and the old home becomes deserted, so much sickness occurring that the place is deemed unhealthful. The causes of this will be found in the sewage-soaked soil, the shade effectually preventing air and sunlight from penetrating its damp recesses. Inspection reveals cellar walls fuzzy with fungi and spores and the rooms smelling musty. The parlor where the city guest was placed, always needed a little fire to take off the chill. The sluggish drain as it meandered into the back yard, had long since become choked with weeds and refused longer to carry the waste from the sink which, unheeded, trickled down the clapboards; the old piece of eavestroughing which aforetime carried it away from the house having long since collapsed. The closet was a rickety old piece of architecture hidden among the clump of lilacs, with vault a one time hollow in the ground and enough to last a century. And near it the well, with its old oaken bucket, reeking with germs. With germs? I hear some one say! Well, only God, in His infinite wisdom, could build a wall of masonry solid enough to protect the progeny of the old time country family against disease and consequent death. Have I drawn this too strongly? I do not think so, for its demonstration is but a question of distance from urban center.

Such conditions as these are unnecessary and without excuse. The thorough disinfection of the soil when surcharged by moisture should be accomplished. Cesspools should be covered with fresh earth after being subjected to sterilising treatment. The old closet should be torn down and burned. A new vault should be excavated at a suitable distance from the well, with sides and bottom cemented to prevent leaking. Proper drainage should be given to the sink, with proper plumbing installed. Old decaying beams and boards should be chopped from cellar walls and ceiling, and replaced with new material. Disinfection of the cellar floor and walls should be effected by whitewashing, and the drain therefrom flushed by antiseptics. A vigorous trimming of the shade trees should let in sunlight and provide for proper air circulation. Suitable windows should be arranged for the free passage of air through the house and cellar. Not until then will the old homestead be fit for human habitation; much less as the place of convalescence for the city sick or the city rest-seeker.

The disposal of sewage in country villages and farm homes is often a very troublesome problem, from the fact of houses often standing on the level, with no fall for the carrying away of waste through pipes. The other fact, that streams flowing through villages belonging to the so-called potable waters of the state, and not being free for use as openings for sewers, makes the problem more intricate still. The slop waters and kitchen refuse is most frequently dumped into the back yard, often a few feet only from the house. Ashes and broken crockery are allowed to accumulate for years. The ash heap often becomes the receptacle for discharges in sickness, until the arrival of a physician pronounces the disease contagious. Even then the discharges are often buried without disinfection. This should never be done without a thorough drenching by antiseptics of the vessel containing such excreta, sputa, or other infected material. After that, burying would be reasonably safe.

Thus, looking to the prevention of sickness, we can by sanitation and careful selection of building sites for homes, eliminate a great many diseases. In choosing a location for a residence we should first examine the adjoining premises as to whether contamination could arise from an unsanitary condition of either of the lots adjoining our prospective site. The one of our selection should have a reasonable slope from the house to the rear, or so arranged that a slight filling could accomplish this.

The greatest attention should be paid to the drainage of the cellar. Proper plumbing for sink and cellar drains should be had, arranging the plan of building to admit western sunlight into as many rooms as possible. It is very necessary that the west wall of the cellar should have one or more large windows where sunlight can be admitted, as without occasional sunlight cellars will become damp and musty. The whole situation may be secured by starting right. No refuse water or slop should be thrown upon the ground. There should be a large galvanised pan made with cover for kitchen refuse which should be transported and burned. Thus, country homes could always be kept pure and maintained under healthful conditions. But the ideal rural home, from a sanitary standpoint, with, of course, some few saving exceptions, is of the future.

Many times when afflicted with some plague, the appeal of the ancients to the Great Spirit would be answered by the sorcerers suggesting a change of residence. The camp having become filthy and germ laden this expedient was often effectual in staying the ravage of the disease. Their possessions being scanty and abandoned at time of removal, most of the infected material was thus left behind. This accounts in large measure for the health of many of the vigorous races of antiquity. When their camps were pitched in a malarial district and ague became common, removal to an elevated position effected a cure of the scourge. So, also, a change of diet would be had in their change of camp from a hunting plateau to the sea shore, where the food would be changed from game to fish.

Another factor in keeping a race inured to such a life, hardy and so physically perfect, was owing to the fact that their mode of life was an exemplification of the survival of the fittest. The disease-affected and consequently weaker part of their population dying off early in life, prevented propagation from other than healthful stock. Thus, we find our Indian, before the appearance of white people, to have been physically an almost perfect race. Their degeneracy is not so much due to disease introduced by the white people as to change in mode of life. Instead of getting the benefit of a change in sanitary conditions following frequent migration, they are huddled together on small reservations and live in badly ventilated houses. The old free life is gone and poor Lo becomes in our time an example of physical degeneracy, from his lack of opportunity in applying his intuitive knowledge of primitive medicine which, in its broad sense, meant to him proper sanitation, diet, climatology and the like. The immunising of animals can be effected, we know, by frequent innoculation with some given disease. So, no doubt, does man become immune from many diseases, by the frequent traversing of his system by the same form of germ life. For instance, those living in malarial districts for a generation will not be affected by the malady, but let a stranger come among them and he is soon stricken.

Wells used for years for drinking purposes by immunised families will not infect them, but a person being a transient visitor to this home, and not accustomed to this water, will soon develop typhoid. This fact would seem to account for the great mortality obtaining among the Indian tribes from tubercular affections. Breeding from healthful stock, their history for ages shows them to have almost, if not quite, weeded out tuberculosis from among them. Their sudden change of habits to a more unsanitary mode of life has led to the devastating inroads of this disease, having secured a habitat in their midst. Their blood containing none of the immunizing serum of previously infected generations, presented virgin soil for the disease and thus they fell an early and sure prey to phthisis.

The sanitation of cities is a much simpler matter than confronts the health officer in the smaller town. Many of these, like my own, are possessed of a water works plant, but are without a system of sewerage. Under these conditions, a vastly greater amount of water is being used than under the old pump system. Consequently the area of soil-contamination becomes vastly greater. Many old wells, cesspools, and such like, being used as outlets for drains, the ground soon becomes impregnated with bacteria. No sewerage being furnished, the best method known to the laity in their germaphobia is to bury the discharges. This is most generally done without disinfection and adds greatly to the unsanitary condition of the water-impregnated soil. In large cities sewerage is easily provided for under the tax system. In smaller towns the rental revenues are not sufficient to withstand such outlay. The consequence is that a sure method of cheaply disinfecting sewage devolves upon the individual, and is very much desired by the health authorities of such places. This disposal of sewage generally takes place upon or near the playground of the village children, and thus becomes a menace to their health in a way not accruing to the city child. The back yard of rural homes being the natural playground of childhood, children are thus subjected to the danger arising from infected ground, and thus their vitality is in constant danger of infection and consequent weakening, at the very period of life when nature is trying to dower them with health for future years. Thus, their playground instead of being an aid becomes a menace.

This suggestion upon prevention of disease might be of the utmost help, but before any radical change comes about in the conditions of the scattered homes of the rural population, there must begin a campaign of education among the masses, without whose coöperation, sanitation is a dead project. Disease will laugh at our efforts. One man may clean up, but his neighbor over the fence flushes against him; and what is he going to do about it? There is no officer to enforce the law; no one wishes to have trouble with the uncouth one who sees no use in being clean, and thinks his neighbor a crank upon sanitary matters.

Prevention of disease, again, is largely a matter of taking care of the children. Especially is this true when looking at the subject from the standpoint of the home.

At the first step we find ourselves confronted by the greatest of modern problems,—the providing of milk which will be suitable for infant life. This is the prime staple of young animal life and unless it be pure the result is not difficult to foresee. Only those who have made a study of the sanitary milk question, can conceive of the appalling mortality directly attributable to its contamination. The transportation of milk by the present methods in cans, which might justly be called giant bacteria-culture-tubes, is an indication of the patience or the stolidness of the public-general. The cleansing of these cans should be done in the city at a sanitary depot by steam or other effective process. Afterward, they should be shipped to the dairy or farm, sealed in such a way as to prevent any possible source of contamination. Our present high rate of mortality, especially among infants, is due to nothing nearly so much as crude methods of sanitation. Trusting a situation demanding the exactest scientific treatment to the hands of an uncultured farmer, whose knowledge of hygiene has never been considered in any age as being above par, and who is often too lazy to do the extra work demanded and too greedy of income to care much for nicety, is sheerest folly. A more careful attention to the care of milk, would safeguard the life and future of the little one against the bacillus coli, one of its greatest enemies. It is tuberculosis, that effective destroyer, that breeds in the filth along our streets, breathes into our faces in every corner, and caught with every gust of wind fills our lungs with his deadly microisms. This is the insidious demon, that until twenty years ago, careered up and down our land secure in his position of being but a hereditary legacy, nine-tenths of the medical profession believing that this malady was not contagious. But now, thanks to the all-seeing eye of the microscope, we know whereof we speak, when we insist that at birth is the proper time to establish the great preventive cordon.

Dairy animals should be tested for tuberculosis before the milk is furnished to the consumer. A tuberculous mother should not be allowed to infect her child. Here is where the education of the masses should begin and such education should be made compulsory. Without the intelligent coöperation of the people, law enactment is of no benefit in the premises. Lectures regarding the transmission of disease should be delivered in public. These should be of frequent occurrence and could be made of great interest. Sanitary classes in public schools should become a part of the curriculum. The training of the children of our time is of the utmost importance here. From their enlightened efforts will come that great crusade against the most direful of all time plagues—tuberculosis. A tuberculous mother who will heedlessly infect her babe against all the warnings, teachings and instructions of this enlightened age, is guilty of a crime and should be treated as a criminal. Therefore, the efforts of the medical profession should be used in one grand effort to make the masses familiar with the means of infection, and thus be on their guard. Here, indeed, is an ounce of prevention worth a pound of cure. This familiarity should include as well the remedies and means to be used against the wholesale spreading of the disease. The laity would assist as assiduously the health officer in the stamping out of tuberculosis as he would in the warfare against diphtheria, provided the hatchery process of tubercle was as short as that of diphtheria. He could then easily reason from cause to effect; but where the effect issues only after possibly years of good, not to say robust health, naturally he loses sight of the cause.

It is in the home, likewise, that habits of personal cleanliness should be inculcated.

A proper care of the teeth and their consequent preservation for the function assigned to them in the physical economy, would go a long way in the prevention of physical disorders. Food, to be assimilated, must be properly ground. Without assimilation the body is not properly nourished and in its weakened state falls an easy prey to disease.

Cases of stomatitis and pyorrhea in children or adults should receive the most careful attention. That this is one of the avenues for the rapid infection of the glandular system is beyond any doubt. An inspection by a physician in school, for instance, will reveal the fact that a large proportion of children have enlarged cervical glands. This will be found to arise from the absorption of matter from carious teeth. How often this matter is loaded with tubercular germs could only be ascertained by the subsequent history of the case. But that the germ of tuberculosis enters the system by this avenue more frequently than by the inhallation process or food infection is, to my mind, an established fact. At all events, the evidence in hand that pus products do enter by this avenue should be the ground for eternal vigilance, that tubercular products be given no opportunity of entering with them.

Then the vessels in daily use by tubercular patients are very rarely cleansed by water which approximates anywhere near to the boiling point, the result being that bacteria adhering to them pass without proper method of destruction. Perishable and easily infected food should be kept in well-ventilated refrigerators, and not in cupboards or cellars. All these things perhaps of seeming unimportance must yet be taken into consideration in the matter of disease transmission.

There is no doubt that substances contaminated with germ life reaches our anatomy by and through the agency of flies. Hence the practice of burying typhoid excreta, or any other sputa or excreta, without proper disinfection is highly reprehensible. The germs from such refuse are undoubtedly brought to the surface of the soil by the heat of the sun, and may then be blown by the wind or carried by the feet of insects to great distances. All discharges should be thoroughly disinfected by the most rigid means before being buried, and thus we would limit the number of our typhoid patients. The contamination of the soil means the contamination of the water supply and consequently the epidemic form of the disease.

The subject of popular medical education as it touches upon the school and school days of man is highly important.

It would not necessitate any great powers of discernment in teachers, nor expert knowledge in the matter of materia medica, to make them competent for detecting the symptoms of many childish ailments. And it would be a very blessed thing if the curriculi of our normal schools provided our teachers with some training, that would allow them to seek out such symptoms in the children committed to their care. Here would be a safeguard and an effectual barrier to the gaining of a foothold of many an epidemic of diphtheria or scarlet fever.

As the child enters school and mingles with its fellows the danger of zymotic infection increases from the crowding at school. Here the ventilation is often abominable, buildings badly heated and always full of dust, although it may be that a school room without dust would be an impossibility while the romping American youth exists. However, a proper disinfection of floors and furniture should frequently be given. This would lesson the number of germs which constantly accumulate in public buildings.

Teachers should be taught to recognise the exanthemata, and a course of instruction put into the curriculum of their normal preparation and instruction, should be given by a physician chosen for that purpose. Many times an epidemic could be prevented and lives saved by an observing teacher who, during the quiet of school hours, would be able to recognise the cough and bleared eyes of measles, or the vomit or flush of scarlet fever. Thus they would learn to send an ailing child from school to a health physician appointed, who would place the child under proper supervision.

All schools should be provided with modern drinking fountains. The old tin cup should be consigned to the age of infection. These fountains are in place in many institutions and allow drinking direct from the jet, without the person touching anything except the stream of water.

It has been proven beyond peradventure that the kissing of infants, and feeding them or administering medicine from the same spoon, are most potent ways of mouth to mouth infection. Great care should be exercised and the fatuous and really meaningless act of kissing a child upon the mouth should be abolished, and its repetition considered to be a crime. The child has no part whatever in kissing. It does not reciprocate the attention though it may unwittingly become the recipient of the most hurtful legacy, which a fond friend or ancestor could bestow. One great mistake of the past was in the belief that people of advanced age did not have tuberculosis, hence grandmother was allowed to kiss and fondle the child with impunity, while a young person might possibly have been barred. It has been abundantly proven that the chronic coughs of old people almost always arise from the action of tuberculosis, and consequently the sputum teems with it. Other diseases besides tuberculosis may be warded off.

In incipient catarrhal conditions great attention should be paid to the condition of the nose and throat. The maintenance of a healthy condition in these two organs, does as much to prevent inroads of pneumonia and diphtheria as any other thing. Enlarged tonsils and adenoids should early receive attention, thus permitting the freest oxygenisation in the lungs of the child. Here again the throat, being free from obstruction and spongy growths, is not so apt to be a prey to bacteria and is more easily cleansed. If diphtheria should occur, it presents less than half the surface for membranous infection. A child presenting the slightest throat symptoms or nasal discharge, should be isolated for a long enough time to be thoroughly cured, and clothing and person disinfected. It is usually the slightest case that passes muster. Thus the disease is spread. No great danger attaches to a bad case of scarlet fever, diphtheria or smallpox because the quarantine will, of necessity, be rigid.

The close affinity of lacunar tonsilitis and inflammatory rheumatism, would indicate that perhaps other germs than those we can demonstrate gain access to the human economy through tonsillar channels. Slight sores in the nose of a child should receive prompt attention, as they often rapidly develop septic symptoms and before one is aware of the gravity of the situation the patient is beyond human aid. The post-nasal space is so enormous, that a wonderful floor space is afforded these germs on which to hold high carnival before one is aware that the dance has begun. A thorough cleansing with peroxide of hydrogen by the nurse or doctor, should be done often during the course of even mild throat lesions. Frequent visits and eternal vigilance should be the rule in cases of this kind.

Ophthalmias and eye lesions should receive the promptest of attention. Their preventive treatment is most exact and its early administration will often sweep away the disease like a cloud. We have only to go to our blind asylums and ask at what age and what the cause of the blindness, to ascertain whether parental education would have lessened the number of dependents in our state institutions. It is safe to say that one third at least would be shut out, and this beautiful world would have been revealed to thousands now groping in hopeless night. What an appeal for popular education is this? Deafness, likewise, in thousands of cases would have been missed, had only the auditory canal been properly cleansed and disinfected in the earlier stage of life. Deafness could be greatly limited by a careful treatment almost entirely to nerve disease.

In conclusion, let us say that this essay is not intended as an exhaustive treatise. It is only suggested in order to awaken enthusiasm in a field practically new, and whose realm is illimitable.

